# Similarity of the cut score in test sets with different item amounts using the modified Angoff, modified Ebel, and Hofstee standard-setting methods for the Korean Medical Licensing Examination

**DOI:** 10.3352/jeehp.2020.17.28

**Published:** 2020-10-05

**Authors:** Janghee Park, Mi Kyoung Yim, Na Jin Kim, Duck Sun Ahn, Young-Min Kim

**Affiliations:** 1 Department of Medical Education, Soonchunhyang University College of Medicine, Asan, Korea; 2 Research and Development, Korea Health Personnel Licensing Examination Institute, Seoul, Korea; 3 Master Center for Medical Education Support, College of Medicine, The Catholic University of Korea, Seoul, Korea; 4 Korea University College of Medicine, Seoul, Korea; 5 Department of Emergency Medicine, College of Medicine, The Catholic University of Korea, Seoul, Korea; 6 Department of Medical Education, College of Medicine, The Catholic University of Korea, Seoul, Korea; Hallym University, Korea

**Keywords:** Educational measurement, Medical education, Medical licensure, Republic of Korea, Reproducibility of results

## Abstract

**Purpose:**

The Korea Medical Licensing Exam (KMLE) typically contains a large number of items. The purpose of this study was to investigate whether there is a difference in the cut score between evaluating all items of the exam and evaluating only some items when conducting standard-setting.

**Methods:**

We divided the item sets that appeared on 3 recent KMLEs for the past 3 years into 4 subsets of each year of 25% each based on their item content categories, discrimination index, and difficulty index. The entire panel of 15 members assessed all the items (360 items, 100%) of the year 2017. In split-half set 1, each item set contained 184 (51%) items of year 2018 and each set from split-half set 2 contained 182 (51%) items of the year 2019 using the same method. We used the modified Angoff, modified Ebel, and Hofstee methods in the standard-setting process.

**Results:**

Less than a 1% cut score difference was observed when the same method was used to stratify item subsets containing 25%, 51%, or 100% of the entire set. When rating fewer items, higher rater reliability was observed.

**Conclusion:**

When the entire item set was divided into equivalent subsets, assessing the exam using a portion of the item set (90 out of 360 items) yielded similar cut scores to those derived using the entire item set. There was a higher correlation between panelists’ individual assessments and the overall assessments.

## Introduction

### Background/rationale

The purpose of the Korea Medical Licensing Examination (KMLE) is to assess whether the test taker possesses the minimum competency needed to hold a medical license. The licensing board has the task of determining appropriate passing criteria. The standard-setting has been carried out to determine the cut score for KMLE clinical skill test since 2009 [1]. However, it was not applied for the written test yet up to 2020. It is necessary to prepare the standard setting methods for the written test also.

Typically, the entire panel determines the final cut score through multiple stages of review of the entire item set [2]. However, this method requires considerable time and effort. Additionally, when panel members are asked to assess a large volume of items, their reliability could be diminished due to fatigue. For these reasons, the licensing board has attempted to implement numerous alternatives for more efficient standard-setting [3]. Ahn et al. [4] in 2018 suggested that the conventional standard setting method, in which all panel members go through multiple rounds of review of the entirety of the item set, is not effective because each item set contains 360 items, which mostly involve problem-solving tasks, making the review process heavily time-consuming, especially as more than 30% of the items have a correct answer rate of 90% or higher.

In general, there are 2 ways to approach this problem: to reduce the number of items each panelist is asked to assess, or to divide the item set into multiple parts for the panel to assess.

In the first approach—reducing the number of items the panel is asked to evaluate—a subset of items needs to be selected from the entire item set. Before this method can be implemented, it needs to be determined that appropriate choices have been made in terms of the appropriate number of items and the selection criteria for items for the selected subset to represent the entire item set adequately. Items can be selected from the item set by random sampling or stratified sampling. In previous research, if the items were randomly selected and if the size of the sample exceeded 50% of the entire item set, the cut score for the sample was similar to the score for the entire item set [5]. When using stratified sampling, panelists considered various properties of the items, including difficulty, discrimination, and content, among which difficulty was most frequently used and highly weighted [6].

Second, panelists can assess the item set by dividing it into a few subsets, which panel members assess individually. Another selection standard is needed to develop each subset, and to do so, we primarily considered the same properties of items as in the stratified sampling process [7].

### Objectives

The purpose of this study was to identify appropriate number of items to set standards more efficiently in the written test of the KMLE, for which the panel on the licensing board is asked to evaluate a large volume of items. We established the following research tasks to achieve these objectives: (1) How many items in each subset would appropriately represent the entire item set? (2) Is there a difference of cut score in test sets with different item amounts? (3) Does the rater reliability change based on the method of assessment according to the number of items?

## Methods

### Ethics statement

This study was approved by the Institutional Review Board of Soonchunhyang University (IRB approval no., 202001-SB-003). Informed consent was obtained from participants (standard-setting panelists).

### Study design

This study involved descriptive analysis and analysis of the panel discussion for the standard-setting of the exam.

### Participants (standard-setting panelists)

The standard-setting panel comprised 15 professors at medical schools in Korea. Considering the subject areas that the examination covers, 2 professors in each of 7 specialties (internal medicine, obstetrics and gynecology, preventive medicine, surgery, pediatrics, psychiatry, and family medicine) and 1 professor in emergency medicine participated. The majority (71.4%) of the panelists had at least 3 years of experience in developing items for national examinations, and 92.8% had at least 5 years of educational experience in universities (Table 1).

### Setting

The examination materials were the KMLE items offered in 2017, 2018, and 2019. Each year examination consists of 60 items on general principles of medical science, 280 items on specific aspects of medical science, and 20 items on medical laws and regulations. A test-taker must achieve 60% or higher on the overall examination and 40% or higher in each test component to pass the examination.

### Composition of subsets

Table 2 presents the procedure of constructing an equivalent subset of the KMLE based on the major area, item discrimination index, and item difficulty index. In the first phase, the major areas were subdivided into 8 specialized areas, and then item discrimination index was categorized as below 0.2 and 0.2 or higher in the second phase. The item difficulty index was classified into 3 categories (below 0.4, between 0.4 and 0.9, and 0.9 or higher) in the third phase. In the fourth phase, the categorized items were labeled A and B, in alternating order, and items with each label were collected in a separate column. In the fifth phase, items were labeled as A1, A2, B1, and B2 in alternating order within each column. Finally, the items with each label (A1, A2, B1, and B2) were grouped together. It was repeated for items of the KMLE 2017, 2018, and 2019.

**Fig. 1.**
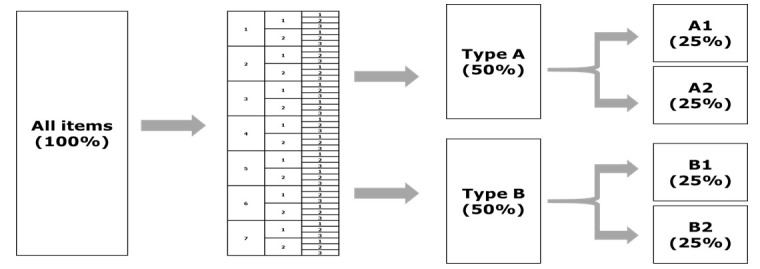
Procedure of classifying the entire item set into subsets based on sorting criteria.

Fig. 1 showed a simple schematic outlining the procedure of categorizing all 360 items. Each grouped item was labeled type A and B based on whether it had an odd or even number in the first phase (50% each). Then we constructed four subsets (type A1, A2, B1, B2) by dividing each group by half. All groups (A1, A2, B1, and B2) were designated as the whole item set (100%). All 15 panelists participated in assessing the whole item set (KMLE 2017). KMLE 2018 and 2019 were assessed by 7 members each (1 from every specialty), in addition to the emergency medicine specialist, who assessed all test items. Therefore, item selection can be said as a stratified sapmpling.

### Standard-setting methods applied

We used the modified Angoff, modified Ebel, and Hofstee methods in this study to set standards. In the modified Angoff method, the panel determined the probability that a marginally competent medical license holder would return the correct answer for each item. We used the average value of the scores submitted by each panel member as the cut score [8]. In the modified Ebel method, the panelists evaluated the relevance of an item and its difficulty. Relevance was assigned as essential, important, or additional knowledge for a license holder beginning the first day of work as a physician. The difficulty level was assigned based on the expected correct answer rate, as easy, medium, or hard [9]. We considered the distribution of the item difficulty index on the KLME when assigning the difficulty level in the modified Ebel method. Easy items had a correct answer rate of 90% or higher, medium items had a rate of 40% to 90%, and hard items had a rate of below 40%. The average value submitted by each panelist for the expected correct answer rate of borderline test-takers was used. In the Hofstee method, each panel member responded with the lowest cut score permissible, the highest cut score, the lowest failure rate, and the highest failure rate. The intersection of the score distribution of test-takers and the values submitted by the panel members served as the cut score [10].

### Implementation process of the standard-setting method

We held standard-setting workshops on February 8 and February 22, 2020, with 2 full-day workshops. On the first day, we introduced the purpose of this research and the standard-setting method and discussed the process of determining the minimum-competency physician. After consensus was reached on the concept of the minimum-competency physician, item sets were provided and assessed. After individual estimations, the panel discussed the results and then derived the final result after a single revision. The detailed schedules of the workshops are presented in Supplement 1. As coronavirus disease 2019 (COVID-19) spread in China, the first workshop was delivered using an in-person model while adhering to quarantine instructions. However, the second workshop was held remotely in light of the rapid spread of COVID-19 in Daegu and Gyeongbuk in Korea. The predetermined schedule was used in the remote session, but the results were confirmed and discussions were held using online methods, including e-mail, messenger (KakaoTalk), and cellphone instant messages.

### Survey for procedural validity

A survey was conducted to ascertain participants’ awareness of the procedure of standard-setting and the results. The survey included 5-point scale items measuring participants’ understanding of the orientation, whether they were comfortable embarking on the assessment process, and whether the respondent believed that the cut score was appropriate. We also collected opinions on assessing the entire item set or assessing a subset of items.

### Statistical methods

Descriptive statistics was used for the assessment results, including the mean differences and confidence levels. We utilized the kappa coefficient for classification accuracy to measure assessors’ reliability. For panel members who assessed 2 subsets of tests, we compared the correlations between their ratings. The correlation coefficients between individual assessment results and the overall results were calculated. The confidence levels for statistical tests were evaluated at the 0.01 level, using IBM SPSS ver. 20.0 (IBM Corp., Armonk, NY, USA).

## Results

### Results for each standard setting method

#### Modifed Angoff method

The final results for the assessment using the modified Angoff method were derived after individual estimation for the selected items in the first round, followed by a second-round estimation after discussion. The cut score for KMLE 2017 was determined to be 63.5% for the first and second rounds. For KMLE 2018, the cut score decreased slightly in the second-round estimation, with 62.0%, compared to 62.8% in the first-round estimation. The cut scores for KMLE 2019 in both rounds of assessment were similar, with 65.3% in the first round and 65.1% in the second round. The results of the standard-setting process using the modified Angoff method are presented in Table 3. The passing rates for each subset were 62.5%, 63.1%, 64.3%, and 64.2%, respectively, which were similar, with an overall passing rate of 63.5%. Subsets A1 and B2 were assessed for KMLE 2018. The passing rates were 62.8% and 61.1%, respectively, with an average of 62%. For KMLE 2019, subsets A1 and B2 were assessed together as a single item set, and the result was 65.1%. The passing rates for each subset were similar. The modified Angoff estimation data of the panel are presented in Dataset 1.

#### Modified Ebel method

The results for the modified Ebel standard-setting process are presented in Table 4. Most items assessed were related to essential knowledge and had medium difficulty. On the percentile scale, the assessment results were 66.4% for KMLE 2017, 67% for KMLE 2018, and 65.7% for KMLE 2019. The data provided by the panel on the expected correct answer rate of the borderline group are shown in Supplement 1.

#### Hofstee method

**Fig. 2.**
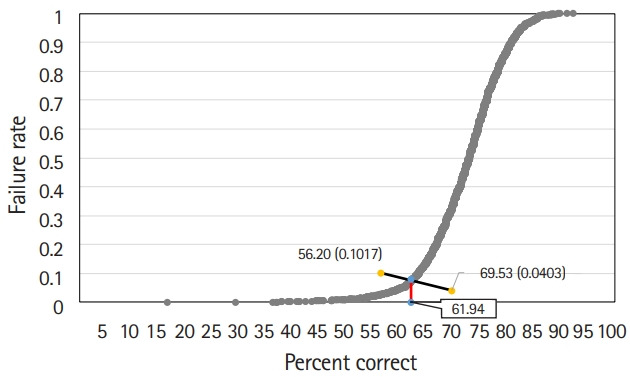
Hofstee graph for the Korean Medical Licensing Examination in 2017.

**Fig. 3.**
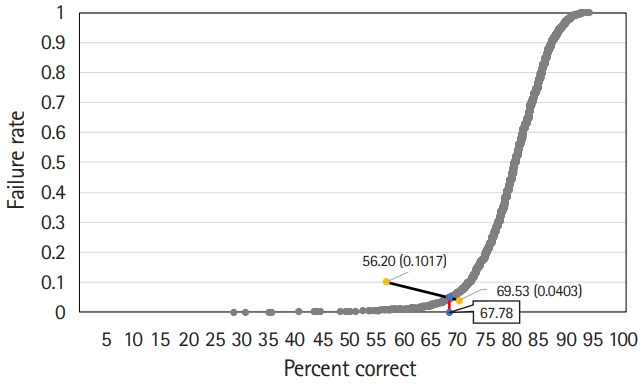
Hofstee graph for the Korean Medical Licensing Examination in 2018.

**Fig. 4.**
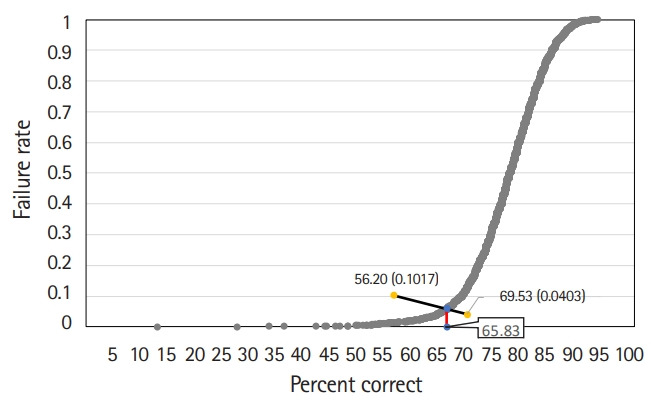
Hofstee graph for the Korean Medical Licensing Examination in 2019.

The Hofstee graph for each test is presented in Figs. 2–4, respectively. On average, the maximum failure rate acceptable for the panel was 10.2%, the minimum failure rate was 4.0%, the highest cut score was 69.5%, and the lowest cut score was 56.2%. The Hofstee assessment results based on the data were 61.9% for KMLE 2017, 67.8% for KMLE 2018, and 65.8% for KMLE 2019 on a percentile scale.

#### Comparison of cut scores between standard-setting methods

The results of the standard-setting process through the modified Angoff, modified Ebel, and Hofstee methods with items from the KMLE from the past 3 years are presented in Table 5.

KMLE 2017 comprised 4 subsets, each containing 25% of the original item set. The average cut score using the modified Angoff method was 63.5%, with a standard deviation of 0.8%; using the modified Ebel method, the average was 66.4%, with a standard deviation of 0.5%; and using the Hofstee method, cut score was 61.9%.

KMLE 2018 comprised 2 subsets, each containing 25% and 26% of the original item set. The average cut score using the modified Angoff method was 62%, with a standard deviation of 0.9%; using the modified Ebel method, the average was 67%, with a standard deviation of 0.5%; using the Hofstee method, cut score was 67.8%.

KMLE 2019 comprised 2 subsets, each containing 51% of the original item set. The average cut score using the modified Angoff method was 65.1%, with a standard deviation of 1.1%; using the modified Ebel method, the average was 65.7%, with a standard deviation of 0.3%; using the Hofstee method, cut score was 65.8%.

The cut score for each standard setting method (modified Angoff, modified Ebel, and Hofstee) was significantly different from that of the other methods for KMLE 2017 (63.5%, 66.4%, and 61.9%, respectively). For KMLE 2017, 100% of the items were assessed. In KMLE 2018, where 51% of the items were assessed, the results of the modified Ebel and Hofstee methods were similar (62%, 67%, and 67.8%, respectively). The results of all standard-setting methods were similar in KMLE 2019, where 51% of the item set was assessed, with 65.1%, 65.7%, and 65.8% for the modified Angoff, modified Ebel, and Hofstee methods, respectively.

### Rater reliability

The inter-rater classification consistency for the modified Angoff method is shown in Supplement 2. The kappa coefficient, which indicates inter-rater classification consistency, was generally high (0.60 or higher) in KMLE 2017. The accuracy of panelists no. 9 and no. 15 was low relative to the other panel members. Notably, panelist no. 9’s assessments did not match those of the other members of the panel at all. The kappa coefficient in KMLE 2018 was very high (0.80 or higher), and the classification consistency between panelists no. 1 and no. 7 was 1.000, indicating identical responses. The kappa coefficient for KMLE 2019 was also generally very high (0.75 or higher), and a value of 1.000 was found between panelists no. 13 and no. 15, indicating another identical match.

The average kappa coefficient in KMLE 2017, excluding panelists no. 9 and no. 15, was 0.72; the average value for KMLE 2018 was 0.92 and that for KMLE 2019 was 0.86. The inter-rater classification consistency in KMLE 2018 and KMLE 2019 was generally higher than that of KMLE 2017.

We calculated the kappa coefficient for measuring the intra-rater classification consistency of each panel member between tests, and the results are shown in Table 6. The intra-rater kappa coefficient between KMLE 2017 and KMLE 2018 was high (0.75), with the exception of panelist no. 9. However, the kappa coefficient between KMLE 2017 and KMLE 2019 varied from 0.56 to 0.92. Excluding panelist no. 9, the classification consistency between KMLE 2017 and KMLE 2018 was higher on average than that between KMLE 2017 and KMLE 2019 (0.88>0.73).

Table 7 shows the results of the paired t-test for the correlation coefficients between individual expected correct answer rates and the average expected correct answer rates. The correlation coefficient between the expected correct answer rate evaluated by individual panelists and the overall average was significantly higher when 50% of the item set was assessed than when the entire item set was assessed.

### Survey results

Table 8 presents the survey results submitted by the panel members. On a 5-point Likert scale ranging from “strongly disagree (1 point)” to “strongly agree (5 points),” the highest score (4.6) was given for the item assessing participants’ understanding of the information given at orientation. The reaction to the general process was favorable. On the question asking whether it was expedient to assume the correct answer rate of the minimum-competency physician, the average score was 3.1, corresponding to the middle of the road. The score for the perceived suitability of the individual assessment grades was 3.6, whereas panelists felt that the determined final cut-off grade was more appropriate, with 4.1 points on average. The response data submitted by the panel members are shown in Dataset 2.

We surveyed participants 3 times regarding the suitability of deriving a standard grade for the KMLE by considering only a subset of the entire item set. Prior to the first workshop, 71.4% of the respondents were favorable to the suggestion, but the rate decreased to 40% right after the second workshop. However, the proportion of favorable responses increased to 50% on the third survey, which was completed after the panel confirmed the overall results for the entire workshop process (Table 9).

We then asked the panelists to list the advantages and disadvantages of developing a cut score with a subset of the item set. The advantages were that it takes less time and effort, as fewer items need to be assessed, and that a more accurate assessment may be possible, as more time is available for discussion since less time is taken up by the assessment process itself. Less time would be consumed by assessing all the items, particularly those with very high correct answer rates. However, panelists were concerned that the licensing board may become vulnerable to potential challenges such as legal action if some of the items are published without an assessment, especially in light of the importance of the exam, which is administered by the state and has major implications for test-takers’ future professional careers. Moreover, some respondents were concerned that it would be challenging to ensure that the selected subset adequately represents the entire item set.

## Discussion

In this research, we examined the utility and reliability of an alternative standard setting method, in which panelists assessed subsets obtained through stratified sampling instead of assessing the entire set of items. Using the item sets that appeared on recent KMLEs, equivalent subsets, each containing around 25% of the original item set, were created based on the item content categories, item discrimination, and item difficulty. The standard-setting results using subsets of 25%, 51%, or 100% of the original item set were analyzed.

### Interpretation

First, when the item set under review was divided into equivalent subsets, and the cut score was derived from some or all of the subsets, the resultant passing rate was highly similar. Of particular note, the smallest subset, which only contained 90 items (25% of the item set), resulted in a similar cut score to that of the estimation process that utilized the entire item set. Ferdous and Plake [3] in 2005 reported that if the size of the subset was 50% or more of the entire item set, the resulting cut score was very similar to the score derived from the entire item set. Kannan et al. [11] in 2015 showed through an analysis using generalizability theory that at least 40 to 50 items were required to achieve estimations with a reliability of 0.80 to 0.90.

Secondly, inter-rater consistency was significantly higher when raters were asked to evaluate 51% of the item set than when they evaluated 100% of the set. Even though we cannot rule out interference from the order effect, as the raters did assess 100% of the item set first, followed by the 51% subset, it is worth noting that the correlation coefficients increased even though the panel members assessed different item set with different pass rates. The panelists responded that the advantages of assessing only part of the item set were that doing so helped to mitigate fatigue, as less time was required for the estimation process, and that reliability increased because more time could be allocated to discussions among the panelists.

Third, we adopted various criteria to select and distribute the items. Previous research employed a random or stratified sampling process to construct a subset from the entire item set. We collected different numbers of items from the entire item set to identify a suitable number. The stratified sampling process utilized multiple properties of items, including major areas, difficulty index, and discrimination index. Items were either selected at random or we jointly considered the properties of each item (item content categories, item discrimination, and difficulty) in our analysis.

Kara and Cetin [12] in 2020 constructed subsets comprising 30%, 40%, 50%, or 70% of the item set based on content areas, difficulty index, and discrimination index, and then analyzed 16 combinations of methods and subtests (4 methods×4 subtests). The most effective method was to develop a subtest with a stratified sample based on the item content categories. This result is also commensurate with those of other previous studies [5,6,12]. We sequentially divided items based on the item content categories, item discrimination index, and item difficulty index. Each group of items was sorted by item number within the item content categories, and then divided evenly between an odd-numbered group and an even-numbered group. It is essential to establish a standard that enables us to allocate the appropriate number of items with a similar distribution every year, reflecting the results of previous examinations, to establish a classification standard for the items on annual examinations in future years. This is the only way to ensure that a suitable number of items are included in each group.

Fourth, based on the survey results from the panelists, even though panel members acknowledged some advantages of only assessing a portion of the items, they were also concerned about the possibility of challenges to the legitimacy of the cut score brought by test-takers who do not pass the exam if the cut score derived from a partial assessment was used in the national examination. Alternatively, it is conceivable to assess the item set based on equivalent subsets instead of a sequential approach to the entire item set, considering the status of the KMLE as a nationally recognized examination. If panelists are asked to assess a higher volume of items, panelist reliability may be diminished as a result of the greater amount of time committed to the assessment and the consequent heightened fatigue of the panelists. In such circumstances, it would be more expedient for the panel to be exposed to equivalent sets of items that they have experience in evaluating, rather than reviewing a new type of item or a new item content category that appears later in the item set while under fatigue.

### Limitations

First, this research only represents a single attempt at carrying out the process described herein. We did not create multiple sets of data by attempting multiple methods of item sampling and partial assessments, which would enable statistical tests of the quantitative analysis for each method itself. Therefore, we cannot definitively conclude that the results from a partial assessment are not different from those obtained by assessing the entire item set. Moreover, it may be very difficult to ensure homogeneity for the properties of items and individual differences among raters. Hence, a simulation may be required to examine whether adjusting some of these conditions also yields the same result. Secondly, we may not have had enough time to cover 3 years’ worth of item sets from the national examination in a 2-day workshop. In particular, the second day of the workshop was held remotely due to the COVID-19 pandemic and may have lacked sufficient discussion compared to those that would have taken place offline. Nonetheless, each panelist recognized the gravity of the situation and engaged in the online workshop actively and professionally.

### Conclusion

In this research, we systematically divided item sets into equivalent subsets as an alternative to the traditional method of standard-setting. The assessments using subsets (25% of whole items) yielded similar cut scores to those of an assessment of the entire item sets, as well as a cut score derived from an assessment procedure using individual subsets. Furthermore, we confirmed that inter-rater consistency was higher when panelists were asked to assess 51% of the items than when panelists were requested to evaluate 100% of the item set. Hence, we believe that this research lies in identifying a basis for a more flexible standard-setting method in the future.

## Figures and Tables

**Table 1.** Characteristics of panel members at medical schools in Korea

Characteristic	No. (%)
Specialty	
Internal medicine	2 (13.3)
Surgery	2 (13.3)
Obstetrics and gynecology	2 (13.3)
Pediatrics	2 (13.3)
Psychiatry	2 (13.3)
Family medicine	2 (13.3)
Preventive medicine	2 (13.3)
Emergency medicine	1 (6.7)
Gender	
Male	10 (66.7)
Female	5 (33.3)
Age group (yr)	
40s	5 (33.3)
50s	8 (53.4)
≥60s	2 (13.3)
Overall	15 (100)

**Table 2.** Classification procedure to obtain equivalent subsets

Step 1: specialty	Step 2: item discrimination index	Step 3: item difficulty index	Step 4: split-half sets 1 (50% for each group)	Step 5: split-half sets 2 (25% for each group)
1	<0.2	0–0.39	A, B, A, B, ...	A1, A2, A1, A2, ...
				B1, B2, B1, B2, ...
		0.40–0.89	A, B, A, B, ...	A1, A2, A1, A2, ...
				B1, B2, B1, B2, ...
		0.90–1	A, B, A, B, ...	A1, A2, A1, A2, ...
				B1, B2, B1, B2, ...
	≥0.2	0–0.39	A, B, A, B, ...	A1, A2, A1, A2, ...
				B1, B2, B1, B2, ...
		0.40–0.89	A, B, A, B, ...	A1, A2, A1, A2, ...
				B1, B2, B1, B2, ...
		0.90–1	A, B, A, B, ...	A1, A2, A1, A2, ...
				B1, B2, B1, B2, ...
2–8				

**Table 3.** The results of the modified Angoff method

Test	Type	No. (%)	Mean %
KMLE 2017	A1	90 (25)	62.5
	A2	90 (25)	63.1
	B1	90 (25)	64.3
	B2	90 (25)	64.2
	Mean		63.5
KMLE 2018	A1	90 (25)	62.8
	B2	94 (26)	61.1
	Mean		62.0
KMLE 2019	A1+B2	182 (51)	65.1
	(A1)	107 (30)	64.1
	(B2)	75 (21)	66.2
	Mean		65.1

KMLE, Korean Medical Licensing Examination.

**Table 4.** The results of the modified Ebel method

Relevance	Difficulty	No. of items			Expected correct answer rate of borderline group	No. of items×expected correct answer rate of borderline group		
		KMLE 2017	KMLE 2018	KMLE 2019		KMLE 2017	KMLE 2018	KMLE 2019
Essential	Easy	2		1	80.67	1.6		0.8
	Medium	215	136	117	69.33	148.4	93.8	80.7
	Hard	2	3	2	53.67	1.1	1.6	1.1
Important	Easy				75.33			
	Medium	135	41	49	63	85.1	25.8	30.9
	Hard		2	2	47.33		0.9	0.9
Additional	Easy				64			
	Medium	6	2	10	48	2.9	1	4.8
	Hard			1	33.47			0.3
Total	-	360	184	182	Cut score	239	123.2	119.6
Score out of 100 points	-					66.4	67	65.7

**Table 5.** Comparison of cut scores

Test	Type	No. (%)	Modified Angoff	Modified Ebel	Hofstee
KMLE 2017	A1	90 (25)	62.5	66	61.9
	A2	90 (25)	63.1	65.9	
	B1	90 (25)	64.3	67.1	
	B2	90 (25)	64.2	66.6	
	Mean±SD		63.5±0.8	66.4±0.5	
KMLE 2018	A1	90 (25)	62.8	66.5	67.8
	B2	94 (26)	61.1	67.4	
	Mean±SD		62.0±0.9	67.0±0.5	
KMLE 2019	A1+B2	182 (51)	65.1	65.7	65.8
	(A1)	107 (30)	64.1	65.9	
	(B2)	75 (21)	66.2	65.4	
	Mean±SD		65.1±1.1	65.7±0.3	

KMLE, Korean Medical Licensing Examination; SD, standard deviation.

**Table 6.** Intra-rater classification consistency (kappa coefficient) for the modified Angoff method

Panelist	KMLE 2017 vs. KMLE 2018	KMLE 2017 vs. KMLE 2019
P1	0.902	-
P2	0.874	-
P6	0.912	-
P9	0.051	-
P10	0.994	-
P11	0.755	-
P14	0.966	-
P7	0.794	0.770
P3	-	0.729
P4	-	0.919
P5	-	0.753
P8	-	0.561
P12	-	0.645
P13	-	0.909
P15	-	0.572

KMLE, Korean Medical Licensing Examination.

**Table 7.** Comparison of correlation coefficients between individual assessments and the overall average

Test	No.	Mean±standard deviation	t-value	Degrees of freedom	P-value
KMLE 2017 vs. 2018			-5.158	6	0.002
KMLE 2017	7	0.519±0.095			
KMLE 2018	7	0.633±0.113			
KMLE 2017 vs. KMLE 2019			-6.072	6	0.001
KMLE 2017	7	0.471±0.071			
KMLE 2019	7	0.636±0.076			

KMLE, Korean Medical Licensing Examination.

**Table 8.** Survey results of the panelists

Test	Question	Mean±standard deviation
Orientation	1. The orientation provided adequate information on the purpose of the standard-setting process for a cut score.	4.6±0.5
	2. The concept of the minimum-competency physician was clear.	4.2±0.8
	3. It was expedient to assume the correct answer rate of minimum-competency physician.	3.1±0.9
	4. I could fill in the assessment form based on the guidelines.	4.4±0.6
KMLE 2017	1. The individual assessment went smoothly.	4.3±0.5
	2. The overall assessment procedure went smoothly.	4.4±0.5
	3. An appropriate rest period was given.	4.4±0.5
	4. The cut score I submitted was appropriate.	3.6±0.6
	5. The final cut score determined by the panel was appropriate.	4.1±0.6
KMLE 2018 and 2019	1. The individual assessments went smoothly.	4.4±0.5
	2. The overall assessment procedure went smoothly.	4.3±0.6
	3. An appropriate rest period was given.	4.5±0.5
	4. The cut score I submitted was appropriate.	3.7±0.6
	5. The final cut score determined by the panel was appropriate.	4.1±0.5

KMLE, Korean Medical Licensing Examination.

**Table 9.** Opinions on determining the cut score by reviewing a subset that represents the entirety of the item set

Response	Before workshop	After workshop	After the result analysis process
Strongly disagree	-	1 (6.7)	-
Disagree	2 (14.3)	6 (40)	2 (20)
Neutral	2 (14.3)	2 (13.3)	3 (30)
Agree	8 (57.1)	5 (33.3)	3 (30)
Strongly agree	2 (14.3)	1 (6.7)	2 (20)
Overall	14 (100)	15 (100)	10 (100)

Values are presented as number (%).

## Footnotes

**Data availability** Data files are available from Harvard Dataverse: https://doi.org/10.7910/DVN/CBOUPU Dataset 1. Panel rating data. Dataset 2. Panel survey data.

## Supplementary materials

Data files are available from Harvard Dataverse: https://doi.org/10.7910/DVN/CBOUPU

Supplement 1. Workshop schedule.

Supplement 2. Inter-rater classification consistency (kappa coefficients) for the modified Angoff method.

Supplement 3. The audio recording of the abstract.
